# Making machine learning matter to clinicians: model actionability in medical decision-making

**DOI:** 10.1038/s41746-023-00753-7

**Published:** 2023-01-24

**Authors:** Daniel E. Ehrmann, Shalmali Joshi, Sebastian D. Goodfellow, Mjaye L. Mazwi, Danny Eytan

**Affiliations:** 1 Department of Critical Care Medicine and Labatt Family Heart Centre, The Hospital for Sick Children, Toronto, ON Canada; 2 Congenital Heart Center at Mott Children’s Hospital and the University of Michigan Medical School, Ann Arbor, MI USA; 3 Center for Research on Computation on Society, Harvard University, Cambridge, MA USA; 4 Faculty of Applied Science and Engineering, University of Toronto, Toronto, ON Canada; 5 Department of Paediatrics, University of Toronto, Toronto, ON Canada; 6 Department of Medicine, Technion, Haifa, Israel

**Keywords:** Health care, Translational research

## Abstract

Machine learning (ML) has the potential to transform patient care and outcomes. However, there are important differences between measuring the performance of ML models in silico and usefulness at the point of care. One lens to use to evaluate models during early development is actionability, which is currently undervalued. We propose a metric for actionability intended to be used before the evaluation of calibration and ultimately decision curve analysis and calculation of net benefit. Our metric should be viewed as part of an overarching effort to increase the number of pragmatic tools that identify a model’s possible clinical impacts.

## Introduction

There is tremendous interest in applying machine learning (ML) to the many unsolved problems in healthcare. Published models can augment clinician awareness, perform diagnostic tasks, forecast clinically relevant events, and guide the clinical decision-making process^1^. However, despite enormous promise and investment, there has been a relatively limited translation of these models to the point of care^2^. This failure of implementation is problematic as it limits the ability to evaluate model efficacy against real-world outcomes.

The reasons for the lack of ML adoption are multifactorial^3^_._ In addition to resource constraints (e.g., lack of data availability, technical infrastructure, certain therapeutic options, and clinical champions), one important barrier to ML adoption may be that many metrics currently used to evaluate and report model performance (e.g., F1 score, area under the receiver operating curve, calibration, discrimination, etc.) don’t reflect how a model would augment medical decision-making^4^. This preoccupation with optimizing traditional performance metrics instead of more clinically applicable ones is a missed opportunity to understand whether a model is likely to be actionable to clinicians faced with a clinical dilemma.

We view actionability as a characteristic of models that reflects their ability to augment medical decision-making when compared to clinician judgment alone. The best single metric measuring a model’s clinical utility is net benefit^5^, which estimates the relationship between a model’s benefits and harms across a range of probability thresholds of decision and disease. Decision curves can be constructed that estimate whether ML or other predictive models would be of higher utility (i.e., net benefit) if acted upon compared to different models or other strategies for testing/treating (e.g., test/treat all patients). If a given model has a higher net benefit compared to alternatives, no matter how much the size of the difference, then the use of the model to make the relevant clinical decision would improve clinical outcomes compared to alternatives. Unfortunately, currently, the net benefit is an underutilized metric, and we feel it should be reported for almost every ML model so readers can better understand its potential utility at the bedside.

However, decision curve analysis is intended to be used on refined models during the final stages of model evaluation. As such, it is not routinely used during early model development. This paper proposes a metric that may aid in identifying a model’s actionability early in development, before evaluation of calibration and ultimately decision curve analysis and calculation of net benefit. Our intent is not to replace traditional metrics of model performance, as they are necessary (but not sufficient) for clinical utility nor replace net benefit. Rather, we view our metric as a clinically oriented filter through which some models should pass early during model development. More broadly, we seek to expand the available tools that holistically evaluate a model’s potential clinical impacts.

## Assessing actionability through the lens of uncertainty

If we define actionability as a characteristic of models that reflects their ability to augment medical decision-making when compared to clinician judgment alone, how might actionable ML augment medical decision-making?

Imagine a clinician must make a diagnosis for a critically ill patient with a fever and then choose an appropriate sequence of treatments based on that diagnosis. Clinicians typically first rank reasonable diagnoses in order of probability in a “differential diagnosis” list based on a complex process of collating, filtering, and weighting data that is often flawed. For example, the clinical history might be incomplete, the physical examination might be unreliable or misleading^6,7^, and tests might be non-specifically abnormal, inaccurate, or non-diagnostic^8^ such that the most probable diagnosis on the differential diagnosis list is wrong up to 40% of the time^9^. Even if the clinician chooses the correct diagnosis or diagnoses, they now must decide on which treatments to prescribe and in which order. Making this determination is often challenged by multiple potential modifiers at the patient level (e.g., the severity of illness, demographics, comorbidities, treatment side effect profiles), provider level (e.g., role, prior training, experience, biases), and system level (e.g., access to certain treatments, cost of treatment). Population-based comparative effectiveness studies and guidelines may provide some guidance, but their application to individual cases can be challenging^10^ and there remains substantial variability in practice and outcomes for even common clinical problems^11^.

Whether at the “diagnosis” or “action” phase of medical decision-making, the clinical dilemma illustrated above is riddled with uncertainty. Excessive uncertainty in medical decision-making is associated with delayed diagnosis^12^, variations in practice^13^, clinician dissatisfaction/anxiety^14^, over testing^15,16^, medical errors^17^, and patient harm^18^. Uncertainty reduction exposes optimal diagnostic or therapeutic choices and removes the friction between competing choices that are associated with either decision paralysis or a “shotgun” approach familiar to many clinicians (where multiple pathways of investigation/treatment are pursued non-specifically and simultaneously, often at increased cost and harm to the patient than a more tailored strategy). Therefore, models that have the tendency to reduce uncertainty in complex clinical scenarios may be valued highly by clinicians, yet there are no ML evaluative metrics specifically designed with this in mind.

## Measuring actionability by quantifying uncertainty reduction

We propose a metric that measures a model’s ability to potentially augment medical decision-making by reducing uncertainty in specific clinical scenarios. Practically, we envision this metric being used during the early phases of model development (i.e., before calculating net benefit) for multiclass models in dynamic care environments like critical care, which are becoming increasingly common in healthcare^19–23^.

To introduce our metric mathematically, we first contend that reducing uncertainty in medical decision-making might mirror the considerations of a partially observable Markov Decision Process (POMDP). In a POMDP framework, the clinician seeks to determine the “correct” diagnosis (in their belief state) and “optimal” treatment by predicting outcomes given a particular action taken. As such, there are two key probability distributions involved: one at the diagnosis phase where the clinician seeks to clarify the distribution of possible diagnoses, and a second at the treatment phase where the clinician seeks to clarify the distribution of future states given actions (i.e., treatments) chosen. Actionable ML should reduce the uncertainty of these distributions.

**Fig. 1:**
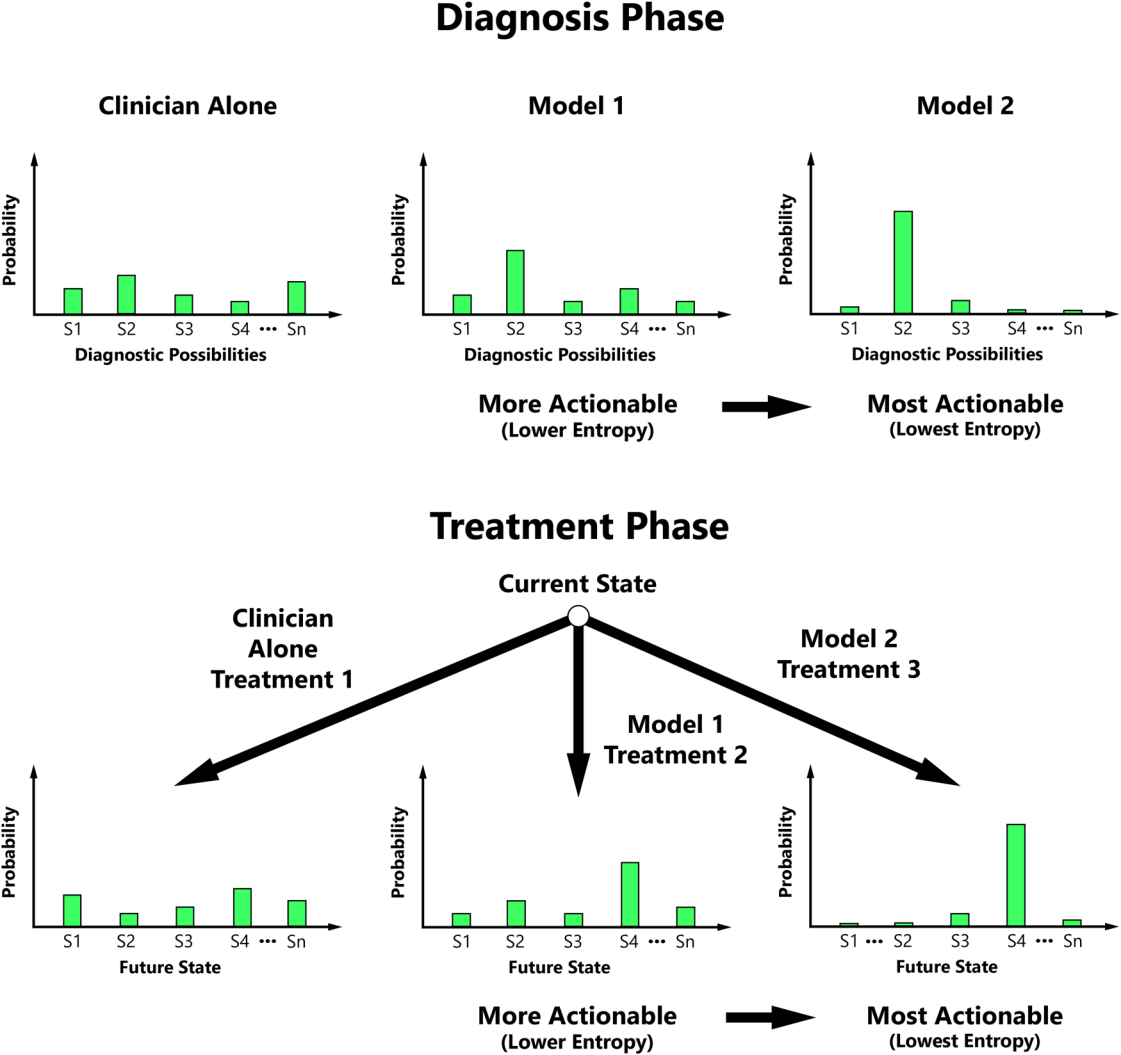
A conceptual schematic illustrating the typical relationship between machine learning actionability and entropy. Actionability typically increases with decreasing entropy of the diagnostic possibility probability distribution and/or conditional future state probability distribution during key phases of medical decision-making. S1 State 1, S2 State 2, S3 State 3, S4 State 4, Sn the Nth State.

The degree of uncertainty reduction in these key distributions can be quantified on the basis of entropy. Entropy is a measurable concept from information theory that quantifies the level of uncertainty for a random variable’s possible outcomes^24^. We propose that clinicians may value entropy reduction, and our actionability metric is therefore predicated on the principle that actionability increases with ML’s ability to progressively decrease the entropy of probability distributions central to medical decision-making (Fig. 1).

Returning to the multiclass model that predicts the diagnosis in a critically unwell patient with fever (among a list of possible diagnoses such as infection, malignancy, heart failure, drug fever, etc.), an ML researcher might use the equation below. The equation is for illustration purposes, acknowledging that additional data are needed to determine the reasonable diagnoses in the differential diagnosis list and their baseline probabilities. This “clinician alone” model might be obtained by asking a sample of clinicians to evaluate scenarios in real-time or retrospectively to determine reasonable diagnostic possibilities and their probabilities based on available clinical data.

For each sample in a test dataset, the entropy of the output from the candidate model (i.e., the probability distribution of predicted diagnoses) is calculated and compared to the entropy of the output from the reference model, which by default is the clinician alone model but can also be other ML models. The differences are averaged across all samples to determine the net reduction in entropy (ML—reference) as illustrated below using notation common to POMDPs:

(1) Clinician Alone Model:

$$H^s_c = - \mathop {\sum}\limits_{s_t \in S} {p_c(s_t|o_t)log\;p_c(s_t|o_t)}$$

(2) With ML Model 1:

$$H^s_{m1} = - \mathop {\sum}\limits_{s_t \in S} {p_{m1}(s_t|o_t)log\;p_{m1}(s_t|o_t)}$$

(3) With ML Model 2:

$$H^s_{m2} = - \mathop {\sum}\limits_{s_t \in S} {p_{m2}(s_t|o_t)log\;p_{m2}(s_t|o_t)}$$

Whereby, $s_t \in S$ is the patient’s underlying state (e.g., infection) at time t within a domain *S* corresponding to a set of all reasonable possible states (e.g., different causes of fever, including but not limited to infection) and $o_t \in O$are the clinical observations (e.g., prior diagnoses and medical history, current physical exam, laboratory data, imaging data, etc.) at time t within a domain *O* corresponding to the set of all possible observations.

Therefore, the actionability of the candidate ML model at the diagnosis (i.e., current state) phase (Δ^**s**^) can be quantified as: $\Delta ^{{{s}}} = {{{H}}}^{{{s}}}_{{{0}}} - {{{H}}}^{{{s}}}_{{{m}}}$, where ${{{H}}}_{{{0}}}^{{{s}}}$ is the entropy corresponding to the reference distribution (typically the clinician alone model, corresponding to ${{{H}}}^{{{s}}}_{{{c}}}$).

Basically, the model learns the conditional distribution of the various possible underlying diagnoses given the observations (see example calculation in supplemental Fig. 1). The extent of a model’s actionability is the measurable reduction in entropy when one uses the ML model versus the reference model.

Continuing with the clinical example above, the clinician must then choose an action to perform, for example, which antibiotic regimen to prescribe among a choice of many reasonable antibiotic regimens. Each state-action pair maps probabilistically to different potential future states, which therefore have a distribution entropy. Acknowledging that additional data are needed to define the relevant transition probabilities $p^ \ast (s_{t + 1}|s_{t,}a_t)$ (i.e., benefit:risk ratios) for each state-action pair (which ideally can be estimated by clinicians or empirically derived data from representative retrospective cohorts) an ML researcher might perform an actionability assessment of candidate multiclass models. The actionability assessment hinges on comparing the entropies of the future state distributions with and without ML and is calculated in a similar fashion to the diagnosis phase, where differences in distribution entropy (reference model - candidate ML model) are calculated for each sample in the test dataset and then averaged. The following equation, or a variation of it, might be used to determine actionability during the treatment phase of care:

Future state probability distribution (P (s_*t*+1_|s_*t*_)

(4) Without ML (e.g., clinician alone action/policy):

$$p_c(s_{t + 1}|s_t) = \mathop {\sum}\limits_{a_t \in A} {p^ \ast (s_{t + 1}|s_{t,}a_t)\pi _c(a_t|s_t)}$$

(5) With ML (e.g., the trained model recommended action/policy):

$$p_m(s_{t + 1}|s_t) = \mathop {\sum}\limits_{a_t \in A} {p^ \ast (s_{t + 1}|s_{t,}a_t)\pi _m(a_t|s_t)}$$

Whereby, *S*_*t*+1_ is the desired future state (e.g., infection resolution), *S*_*t*_ is the current state (e.g., fever) at time *t*, $a_t \in A$ is the action taken at time *t* within a domain *A* corresponding to a set of reasonable possible actions (i.e., different antibiotic regimens), $\pi _c(a_t|s_t)$ is the policy chosen by the clinician at time *t* (e.g., treat with antibiotic regimen A) and $\pi _m(a_t|s_t)$ is the policy recommended by ML at time *t* (e.g., treat with antibiotic regimen B).

Entropy (*H*) of the future state probability distribution

Each future state probability distribution comes from a distribution of possible future states with associated entropy, which we illustrate as:

(6) Without ML:

$$H^a_0 = - \mathop {\sum}\limits_{s_{t + 1} \in S} {p_0(s_{t + 1}|s_t)log\;p_0(s_{t + 1}|s_t)}$$

(7) With ML:

$$H^a_m = - \mathop {\sum}\limits_{s_{t + 1} \in S} {p_0(s_{t + 1}|s_t)log\;p_m(s_{t + 1}|s_t)}$$

Therefore, the actionability of the candidate ML model at the action (i.e., future state) phase (Δ^*a*^) can be quantified as $\Delta ^{{{a}}} = {{{H}}}^{{{a}}}_0 - {{{H}}}^{{a}}_{{{m}}}$, where ${{{H}}}_0^{{{a}}}$ is the entropy corresponding to the reference distribution (typically the clinician alone model).

The model essentially learns the conditional distribution of the future states given actions taken in the current state, and actionability is the measurable reduction in entropy when one uses the ML model versus the reference (typically clinician alone) model.

## Conclusions

Despite the tremendous promise, the adoption of ML for challenging decisions in clinical practice remains relatively limited. A key objective of this paper was to participate in a robust conversation about what pragmatic metrics might help to evaluate clinical utility more directly than traditional metrics of model performance. The net benefit is the single best metric to evaluate a model’s clinical impact at the bedside and it should be measured and reported with greater frequency than currently found in the ML literature. However, decision curve analysis should be performed during the final stages of model evaluation, and we propose another tool designed for early model development that may have value to clinician end-users of ML designed for complex, multiclass algorithms in dynamic care environments. When hundreds of models or parametrizations of models are being calibrated or tuned, we believe that researchers might apply another clinically oriented filter by asking: to what extent might candidate models be actionable, that is, augment medical decision-making when compared to clinician judgment alone? We argued that actionability might be related to uncertainty reduction and that uncertainty reduction could be measured using entropy applied to key probability distributions in a POMDP framework.

The actionability and entropy reduction framework is not perfect, and we acknowledge important limitations that preclude its use later during model development (i.e., when net benefit should be calculated) or in isolation. For example, we acknowledge that a model that fails to significantly reduce entropy may not necessarily lack clinical utility and conversely, a model that significantly reduces entropy but otherwise performs poorly may just be confidently wrong. Furthermore, uncertainty reduction is likely important to medical decision-making, but the line connecting uncertainty reduction and medical decision-making is not absolute and linear but rather imperfect. For example, even robust models that lower entropy relative to other models or clinician judgment alone may not be actionable for a variety of reasons (e.g., a lower entropy differential diagnosis may not change the testing approach, a lower entropy future state distribution may not be clinically modifiable and thus no action is performed) and some models that increase entropy may be actionable for a variety of reasons (e.g., models that “screen” for diagnoses and introduce more uncertainty by appropriately increasing the probability of diseases for patients felt to be at low baseline risk). We acknowledge that our metric may also be impacted by our arbitrary choice of targeting entropy reduction of future state distributions when others may have targeted entropy reduction of different policies. Last, our metric may be sensitive to modeling techniques that impact the complexity of probability distributions. A simple model that always predicts the probability of a given diagnosis at 100% (and other reasonable diagnoses at 0%) will appear to have more actionability using the proposed framework than a more complicated model that covers a wider range of the probability space. Given these limitations, we reiterate that our actionability metric should be considered a tool that may help shed light on which models should proceed to more rigorous evaluation prior to net benefit calculation and eventual bedside deployment.

More broadly and importantly, our focus was not on proposing the only, perfect, or best way measure actionability nor that actionability assessments should replace existing metrics, such as net benefit. Rather, we seek to foster necessary conversations on the importance of model actionability. We hoped to introduce important elements of that conversation by profiling the importance of uncertainty in medical decision-making for clinical problems and environments that will inevitably be the topic of continued investigation in the ML community. We propose that measuring a model’s ability to get it “right” or “wrong” (i.e., true positives, false positives, etc.) is important, but so is a way to understand the consequences of that ability (i.e., net benefit) and the distribution of outputs in multiclass ML through the lens of uncertainty and medical decision-making. Our approach, or a similar approach, might spur important lines of investigation for the field. For example, can quantifying uncertainty reduction augment net benefit calculations in the special case of complex multi-class problems? Might ML teams more frequently evaluate project proposals through the lens of uncertainty and allocate greater resources to problems that induce more clinical uncertainty over those that induce less clinical uncertainty? Further research will be required and should be actively encouraged.

A future characterized by greater emphasis on model actionability might not be imminent, but we provide one suggestion for progress in that direction. A wider armamentarium of tools to evaluate a model’s potential impacts at the bedside will be required to unleash the power of ML for the benefit of clinicians and patients.

### Reporting summary

Further information on research design is available in the Nature Research Reporting Summary linked to this article.

## Supplementary information

Supplemental Material

Reporting Summary Checklist
