# Supplementary material for: Making machine learning matter to clinicians: model actionability in medical decision-making
Source: NPJ Digit Med. 2023 Jan 24;6:7. doi: 10.1038/s41746-023-00753-7 (PMC9871014; doi:10.1038/s41746-023-00753-7)
Supplement: Supplementary file 1 — Supplemental Material [file 41746_2023_753_MOESM1_ESM.pptx]

## Slide 1
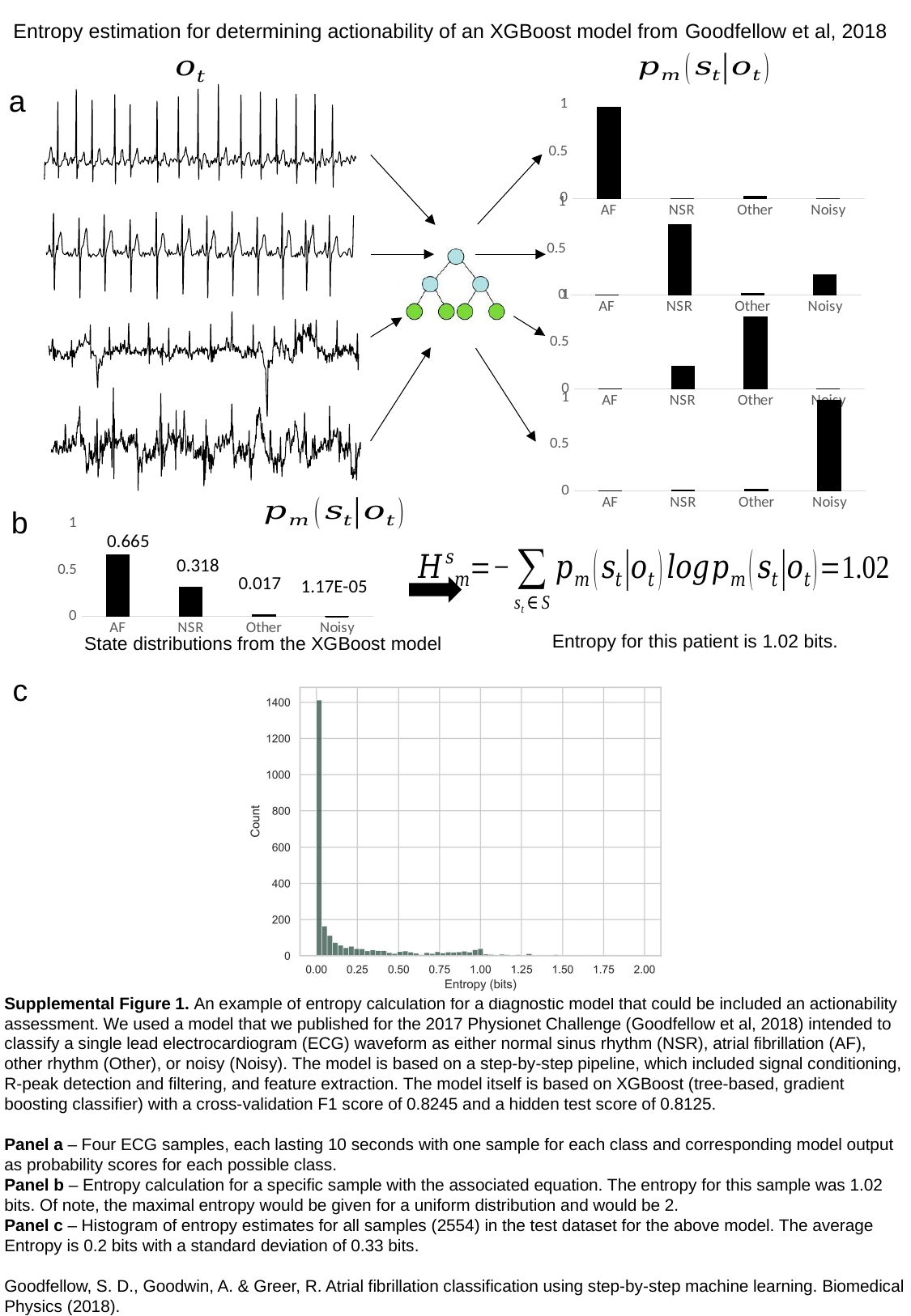

Entropy estimation for determining actionability of an XGBoost model from Goodfellow et al, 2018
a
### Chart
| Category | |
|---|---|
| AF | 0.9720857 |
| NSR | 7.157013e-07 |
| Other | 0.027912091 |
| Noisy | 1.4664579e-06 |
### Chart
| Category | |
|---|---|
| AF | 0.00097307016 |
| NSR | 0.76130486 |
| Other | 0.020797731 |
| Noisy | 0.21692432 |
### Chart
| Category | |
|---|---|
| AF | 7.351709e-05 |
| NSR | 0.23682496 |
| Other | 0.7630773 |
| Noisy | 2.4210764e-05 |
### Chart
| Category | |
|---|---|
| AF | 0.00027306244 |
| NSR | 0.0060977186 |
| Other | 0.013157538 |
| Noisy | 0.9804717 |0.318
0.017
1.17E-05
Entropy for this patient is 1.02 bits.
State distributions from the XGBoost model
b
0.665
c
Supplemental Figure 1. An example of entropy calculation for a diagnostic model that could be included an actionability assessment. We used a model that we published for the 2017 Physionet Challenge (Goodfellow et al, 2018) intended to classify a single lead electrocardiogram (ECG) waveform as either normal sinus rhythm (NSR), atrial fibrillation (AF), other rhythm (Other), or noisy (Noisy). The model is based on a step-by-step pipeline, which included signal conditioning, R-peak detection and filtering, and feature extraction. The model itself is based on XGBoost (tree-based, gradient boosting classifier) with a cross-validation F1 score of 0.8245 and a hidden test score of 0.8125.
Panel a – Four ECG samples, each lasting 10 seconds with one sample for each class and corresponding model output as probability scores for each possible class.
Panel b – Entropy calculation for a specific sample with the associated equation. The entropy for this sample was 1.02 bits. Of note, the maximal entropy would be given for a uniform distribution and would be 2.
Panel c – Histogram of entropy estimates for all samples (2554) in the test dataset for the above model. The average Entropy is 0.2 bits with a standard deviation of 0.33 bits.
Goodfellow, S. D., Goodwin, A. & Greer, R. Atrial fibrillation classification using step-by-step machine learning. Biomedical Physics (2018).
